# Post-transplant cyclophosphamide for GVHD prophylaxis in pediatrics with chronic active Epstein-Barr virus infection after haplo-HSCT

**DOI:** 10.1186/s13023-022-02585-2

**Published:** 2022-12-02

**Authors:** Rongmu Luo, Xiaomei Zhang, Ya Wang, Qihang Man, Wenjing Gu, Zhengqin Tian, Jingbo Wang

**Affiliations:** 1 Department of Hematology, Aerospace Center Hospital, No. 15, Yuquan Road, Haidian District, Beijing, 100049 China; 2 Department of Hematology, Senior Department of Pediatrics, The Seventh Medical Center of PLA General Hospital, Beijing, 100700 China

**Keywords:** Chronic active Epstein-Barr virus infection, Hematopoietic stem cell transplantation, Anti-thymocyte globulin, Post-transplant cyclophosphamide, Graft-versus-host disease prophylaxis

## Abstract

**Background:**

Chronic active Epstein-Barr virus infection (CAEBV) is a rare but life-threatening progressive disease. Human leukocyte antigen (HLA)-haploidentical hematopoietic stem cell transplantation (haplo-HSCT) is the best choice as sometimes HLA-matched donor is not accessible. However, graft-versus-host-disease (GVHD) following transplantation remains a major cause of treatment failure and elevated mortality. Post-transplant cyclophosphamide (PTCy) has recently emerged for effective GVHD prophylaxis in a haploidentical setting in many hematologic malignancies. Here, we report the performance of PTCy for GVHD prophylaxis in a series of CEABV patients treated with haplo-HSCT.

**Methods:**

Consecutive pediatric CAEBV patients who were treated with haplo-HSCT and give PTCy for GVHD prophylaxis were analyzed. 1-year GVHD and relapse-free survival (GRFS), overall survival (OS) and cumulative incidence of moderate-to-severe chronic GVHD (cGVHD) were estimated.

**Results:**

A total of 8 patients ranging from 2 to 15 years old were included. Among them, 4 patients had early complications after haplo-HSCT. Counts of T-cell subsets increased within 6 months post transplantation, indicating an immune reconstitution. Only 1 patient developed grade II acute GVHD, and 2 patients had moderate cGVHD. One patient died from diffuse alveolar hemorrhage within the first year after transplantation. The 1-year GRFS rate, OS rate and cumulative incidence of moderate-to-severe cGVHD were 62.5%, 87.5% and 25.0%, respectively.

**Conclusion:**

Our findings suggest that, among CAEBV patients treated with haplo-HSCT, PTCy may be an alternative choice for the prevention of GVHD.

## Background

Primary Epstein-Barr virus (EBV) infection mainly invades B lymphocytes and is usually asymptomatic in children and can cause the self-limiting infectious mononucleosis (IM) in adolescents and adults [1]. Occasionally, EBV infects T cells and natural killer (NK) cells, leads to persistent proliferation and infiltration of these EBV-infected cells into multiple organs, which finally causes lymphoproliferative diseases (LPD), such as chronic active Epstein-Barr virus infection (CAEBV) [2]. CAEBV is rare but may be life-threatening. The clinical manifestations are mainly persistent IM-like symptoms, including fever, interstitial pneumonia, persistent lymphadenopathy, splenomegaly and impaired liver function [3]. In the absence of treatment, this progressive disease develops with severe complications, such as opportunistic infections, multi-organ failure, hemophagocytic lymphohistiocytosis (HLH) and lymphoma [4, 5] and has a high mortality rate. Kimura et al. reported that 35 (42.7%) out of 82 CAEBV patients died within 5 months to 12 years after onset of CAEBV [6]. The high mortality rate may be attributed to late diagnosis and treatment given to most patients, and a low percentage of patients receiving the curative hematopoietic stem cell transplantation [6].

CAEBV is refractory to antiviral therapy, immunosuppressive therapy, cytotoxic drug-based chemotherapy or cytotoxic T lymphocytes (CTL) [3]. Till today, allogeneic hematopoietic stem cell transplantation (allo-HSCT) is the only curative treatment for CAEBV [7–10]. However, a considerable proportion of patients after allo-HSCT develop chronic graft-versus-host-disease (cGVHD) resulting in elevated mortality and reduce quality of life [11, 12]. It has been reported that almost a half of patients develop cGVHD within 36 months after allo-HSCT [13]. Analysis on a large cohort of patients treated with myeloablative allo-HSCT revealed significant association between cGVHD and higher risk of treatment-related mortality and inferior overall survival [11]. Thus, GVHD remains a major impediment to the wide applicability of HSCT.

Currently, the combination of calcineurin inhibitor (CNI) and methotrexate (MTX) is standard for acute GVHD (aGVHD) prophylaxis, and the addition of anti-thymocyte globulin (ATG) to standard protocol has exhibited good performance and been recommended for the prevention of GVHD [14, 15]. These regimens are commonly used in human leukocyte antigen (HLA)-identical sibling or unrelated donor transplant. yielding a low rate of GVHD [16]. Whereas, similar performance in mismatched donor transplant has not been expected. It’s unfortune that only a small proportion of patients have HLA-matched siblings [17] and the proportion is even smaller in China due to the one-child policy implemented in recent decades. HLA-haploidentical related donor is an alternative source of stem cells [17], which is the main source of HSCT among Chinese CAEBV patients. An effective strategy for GVHD prophylaxis in HLA-haploidentical donor transplant is warranted.

High dose of post-transplant cyclophosphamide (PTCy) has emerged as an effective method for GVHD prophylaxis in HLA-haploidentical donor transplantation (haplo-HSCT) [18]. Large-scale analyses demonstrated acceptable rates of acute and chronic GVHD and long-term survival in this group of patients [19, 20]. In addition, the applicability of PTCy in non-haplo setting, such as matched related donor (MRD) and matched unrelated donor (MUD) transplants, has also been explored, showing favorable results in terms of preventing GVHD and improving survival [21, 22]. However, there are few reports on PTCy for GVHD prophylaxis in CAEBV cases who were treated with haplo-HSCT. Here, we reported a series of pediatric CAEBV patients who were given PTCy for GVHD prophylaxis after haplo-HSCT.

## Methods

### Patients

Consecutive pediatric patients who were diagnosed with CAEBV and received haplo-HSCT at two transplant centers, the Aerospace Center Hospital and the Seventh Medical Center of PLA General Hospital, Beijing, China, between 2019 and 2020, were included. CAEBV was diagnosed according to the following criteria [23, 24]: (1) persistent or recurred infectious mononucleosis (IM)-like symptoms for more than 3 months; (2) evidence of EBV infection and histopathological damage; (3) excluding primary infection of EBV, autoimmune diseases, tumor diseases and immunodeficiency diseases. The IM-like symptoms included fever, swollen lymph nodes and hepatosplenomegaly, pancytopenia, complications in digestive tract (e.g. bleeding and ulcers), lung (such as interstitial pneumonia), eyes (such as retinitis), skin (such as hydroa vacciniforme and mosquito allergy) and cardiovascular (including aneurysm and valvular disease). The EBV infection should fulfill two of the following three conditions: (1) EBV-DNA level in peripheral blood mononuclear cell (PBMC) is higher than 10^2.5^ copies/µg DNA, or the serum/plasma EBV-DNA is positive; (2) Epstein-Barr Virus-Encoded Small RNAs (EBERS) in situ hybridization or Epstein-Barr Virus latent membrane protein 1 (EBV-LMP1) immunohistochemical staining is positive in the affected tissue; (3) Southern blotting detecting EBV-DNA in tissues or peripheral blood.

This study was approved by the ethical committees of the Aerospace Center Hospital and the Seventh Medical Center of PLA General Hospital and performed according to the Declaration of Helsinki. Written informed consents were given by all participants and/or their parents.

### EBV-infected cells

PBMCs were divided into CD3+ cells, CD19+ cells, and CD56+ cells using Magnetic beads. Patients were defined as T-cell type when CD3+ cells were the major group of EBV-infected cells (EBV DNA in T cells > 1 × 10^3^ copies/mL) and as NK-cell type when CD56+ cells were the major group (EBV DNA in NK cells > 1 × 10^3^ copies/mL). If both CD3+ cells and CD56+ cells were the major group, they were defined as combined T and NK cell type (T/NK-cell type). All patients underwent pathological biopsy of lymph nodes or bone marrow. The EBV copy numbers were measured by real-time quantitative PCR.

### Treatment before HSCT

All patients were divided into active or non-active CAEBV when admitted to hospital according to clinical features and EBV load. Active disease was defined as the presence of the following symptoms and signs: fever, persistent hepatitis, lymphadenopathy, hepatosplenomegaly, pancytopenia and/or progressive skin lesions, accompanied by increased peripheral blood EBV-DNA load. Otherwise, it was classified as non-active disease.

Non-active patients were given 2–3 cycles of modified CHOP regimen (cyclophosphamide, doxorubicin, vincristine and prednisolone) and then received planned HSCT. Active CAEBV patients received 2 weeks of REP (ruxolitinib, etoposide, methylprednisolone) treatment. If non-active disease was achieved, planned HSCT was executed after 2–3 cycles of modified CHOP regimen. For patients who developed hemophagocytic syndrome or resistance to the REP protocol, the DEPL (doxorubicin hydrochloride liposome, etoposide, methylprednisolone, L-asparaginase or pegaspargase) regimen were administered. After all, persistent-active cases were given emergency HSCT.

### Conditioning regimen and GVHD prophylaxis

All patients received myeloablative conditioning (MAC) regimen before haplo-HSCT, which included fludarabine (40 mg/m^2^/day on days − 10 to − 7), etoposide (100 mg/m^2^/day, days − 10 to − 8), busulfan (3.2–4.8 mg/kg/day on days − 7 to − 4) and cyclophosphamide (14.5 mg/kg/day on days − 3 to − 2). For the purpose of GVHD prophylaxis, patients were given PTCy. The GVHD prophylaxis program used cyclosporine/tacrolimus (starting on day + 5), short-term MTX (15 mg/m^2^ on day + 1 and 10 mg/m^2^ on day + 11), mycophenolate mofetil (30 mg/kg/day divided into two oral doses, starting on day + 5) and cyclophosphamide (50 mg/kg/day on days + 3 and + 4).

### Post-transplant evaluation

Acute GVHD (aGVHD) was assessed and graded according to the consensus criteria [25]. Chronic GVHD (cGVHD) was scored according to the 2014 NIH consensus criteria and the severity was categorized as mild, moderate or severe [26]. GVHD and relapse-free survival (GRFS) was defined as the first occurrence of III−IV aGVHD, moderate-to-severe cGVHD, relapse or death of any cause after HSCT [22].

### Analysis of lymphocyte subsets after HSCT

The lymphocyte subsets, including CD3+, CD4+, CD8+ T cells, NK cells and B cells, in peripheral blood were analyzed by flow cytometry at 3, 6 and 12 months after HSCT.

### Statistical analysis

Continuous variables were presented as median and range, and paired data were compared using nonparametric Wilcoxon signed rank test. Categorical variables were presented as number and percentages. 1-year cumulative incidence of moderate-to-severe cGVHD, 1-year GRFS rate and 1-year OS rate were estimated. All statistical analyses were performed by using PRISM 8 (GraphPad Software, US) and STATA 12.0 (Stata Corporation, USA).

## Results

### Patients characteristics

A total of 8 pediatric CAEBV patients who received PTCy for GVHD prophylaxis after haplo-HSCT were included in analysis. There were 3 males and 5 females. The median age at onset was 4.5 years (range 2–15) and the majority were younger than 8 years. The median time from diagnosis to HSCT was 6.5 months (range 4–87). Most of the patients were T or T/NK-cell type and only 2 was NK-cell type with regard to EBV-infected cells. At transplantation, 4 patients were at active disease status and 4 were non-active disease status. All donors were HLA-haploidentical parents of recipients (i.e. haplo donor). All patients used peripheral blood as graft source. The majority had various complications before HSCT, including herpes zoster, sepsis, rash and so on. The median counts of infused mononuclear cell (MNC) and CD34+ T cell were 10 × 10^8^/kg (range 6.05–17.05) and 7.73 × 10^6^/kg (range 3.43–17.67), respectively. The clinical characteristics all patients were summarized in Table 1.

**Table 1:** Baseline and clinical characteristics of all CAEBV patients

Patient	Age at onset, years	Gender	Complication before HSCT	Stem cell source (donor, HLA identity)	Donor type	Time to HSCT, months	Disease status	EBV-infected cells	Preconditioning	Infused cells	
										MNC (10^8^/kg)	CD34+ (10^6^/kg)
PTCy-1	15	M	Herpes zoster	PB (father, 7/10)	Haplo	87	Active	NK	FLU/VP16/BU4/CY	6.05	3.43
PTCy-2	4	F	None	PB (father, 7/10)	Haplo	4	Non-active	T	FLU/VP16/BU4/CY	7	10
PTCy-3	4	M	Sepsis, lung infection	PB (father, 5/10)	Haplo	16	Active	T/NK	FLU/VP16/BU4/CY	17.05	17.67
PTCy-4	2	M	Virus pneumonia; central invaded	PB (father, 5/10)	Haplo	5	Non-active	T/NK	FLU/VP16/BU4/CY	14	8
PTCy-6	3	F	Pancreatitis; enlarged coronary artery	PB (father, 5/10)	Haplo	6	Active	T/NK	FLU/VP16/BU4/CY	10	7.46
PTCy-7	6	F	Myocardial damage, cardiomegaly	PB (mother, 5/10)	Haplo	37	Active	T/NK	FLU/VP16/BU4/CY	16.8	6.9
PTCy-8	6	F	Papular rash	PB (father, 7/10)	Haplo	7	Non-active	T/NK	FLU/VP16/BU4/CY	10	8
PTCy-9	5	F	Rash	PB (mother, 5/10)	Haplo	6	Non-active	NK	FLU/VP16/BU4/CY	10	6.8

CAEBV: chronic active Epstein-Bar virus infection; PTCy: post-transplant cyclophosphamide; HSCT: hematopoietic stem cells transplant; HLA: human leukocyte antigen; PB: peripheral blood; BM: bone marrow; NK: natural killer; Haplo: haploidentical donor; MSD: matched sibling donor; MNC: mononuclear cell; M: male; F: female

### Early complications, infections and viral reactivation after transplants

We documented the occurrence of complications, infections and viral reactivation within the first years after transplants (Table 2). Early complications were not observed in 4 patients. Of the remaining patients, 1 developed hemorrhagic cystitis (HC) and reversible posterior leukoencephalopathy syndrome (RPLC), 1 had immunologic lung injury, 1 developed engraftment syndrome (ES) and poor graft function (PGF), and 1 had drug-induced seizure. Early infections were found in 3 patients, including 1 bacterial infection and 2 cytomegalovirus (CMV) retinitis. CMV was reactivated in 6 cases and EBV was reactivated in 2 patients.

**Table 2:** Clinical outcomes of all CAEBV patients post HSCT.

Patient	aGVHD location^#^	cGVHD location^$^	Early complications	Early infections	Viral reactivation	Survival	Cause of death
PTCy-1	None	None	HC, RPLC	Sepsis, urinary system infection (bacteria)	CMV	Dead	Diffuse alveolar hemorrhage
PTCy-2	Intestine (II)	None	Immunologic lung injury	CMV retinitis	CMV	Alive	–
PTCy-3	None	Digestive tract (M)	None	None	EBV	Alive	–
PTCy-4	None	None	None	None	CMV	Alive	–
PTCy-6	None	None	None	None	None	Alive	–
PTCy-7	None	None	ES, PGF	CMV retinitis	CMV	Alive	–
PTCy-8	None	Lung (M)	Drug-induced seizure	None	CMV, EBV	Alive	–
PTCy-9	None	None	None	None	CMV	Alive	–

^#^Only grade II-IV aGVHD were listed

^$^Only moderate (M) and severe (S) cGVHD were shown

aGVHD/cGVHD: acute/chronic GVHD; HC: hemorrhagic cystitis; RPLS: reversible posterior leukoencephalopathy syndrome; PGF: poor graft function; ES: engraftment syndrome. CMV: cytomegalovirus; EBV: Epstein-Bar virus

### Immune reconstitution

**Fig. 1:**
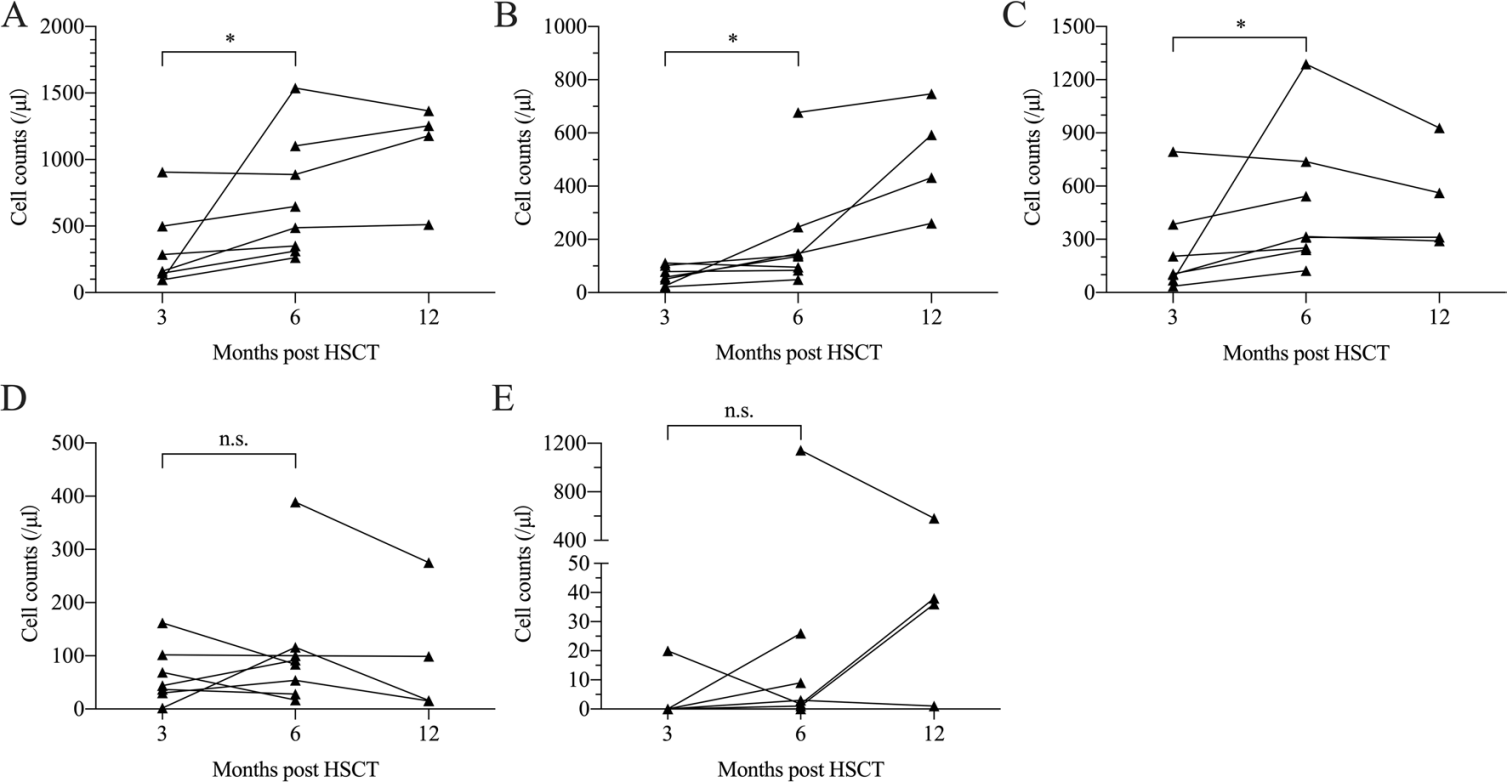
Immune reconstitution of CAEBV patients at 3, 6 and 12 months after haplo-HSCT. **A** CD3+ T cells; **B** CD4+ T cells; **C** CD8+ T cells; **D** NK cells; **E** B cells. CAEBV: chronic active Epstein-Barr virus infection; haplo-HSCT: HLA-haploidentical hematopoietic stem cell transplantation; Paired data was compared with Wilcoxon signed rank test. *: *p* < 0.05; n.s.: not significant

Paired test using Wilcoxon signed rank test showed significant increase of CD3+, CD4+ and CD8+ T cells from 3 months to 6 months after transplantation, indicating a recovery of T-cell subsets and immune reconstitution (Fig. 1 A–C). Counts of NK cell and B cell did not significantly differ between 3 months and 6 months after HSCT (Fig. 1D, E). Between 6 months and 12 months post HSCT, counts of CD4+ T-cell subset continued to increase, but there were no significant changes of the other subsets (Fig. 1).

### GVHD and survival

**Fig. 2:**
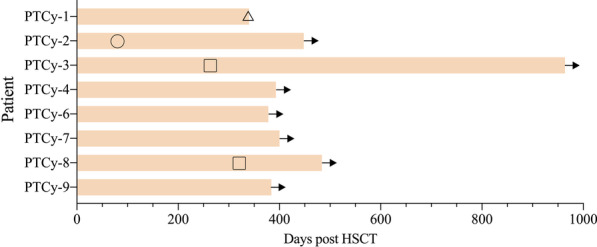
Schematic plots of survival and GVHD events of each CAEBV patient after haplo-HSCT. No relapse was observed. Triangle: death; circle: acute GVHD; square: chronic GVHD; arrow: alive

**Fig. 3:**
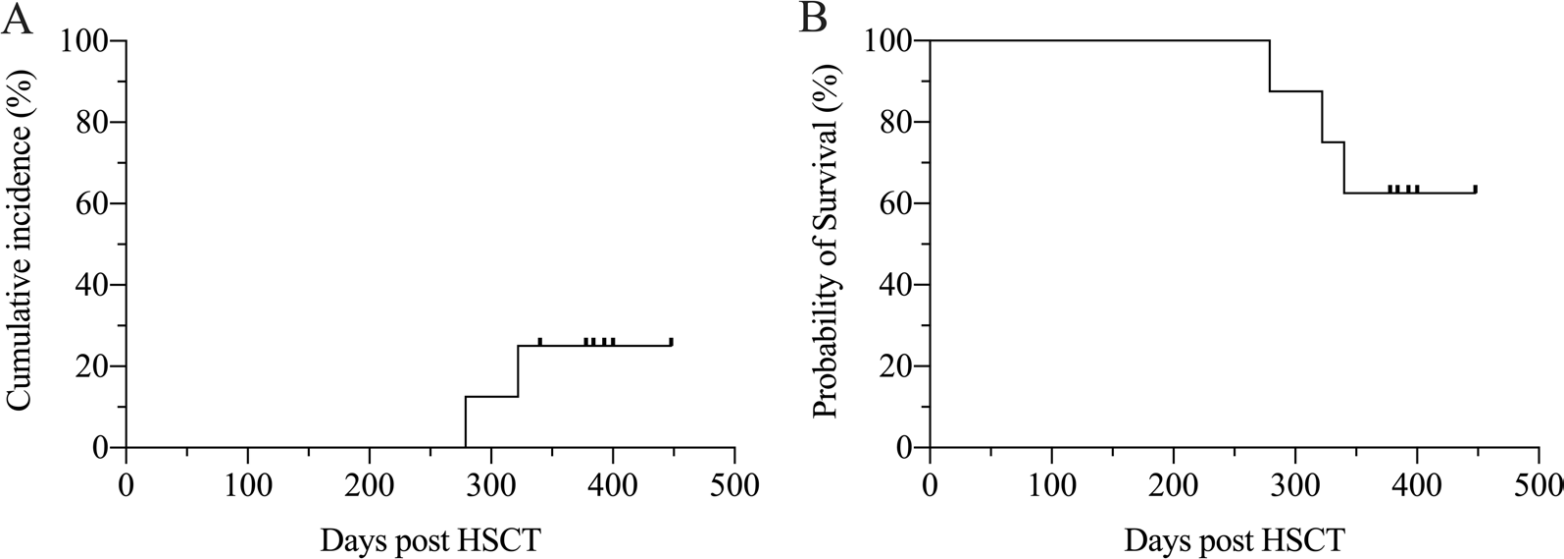
Cumulative incidence curve for moderate-to-severe cGVHD (**A**) and Kaplan–Meier curve for GRFS (**B**) in CAEBV patients receiving PTCy after haplo-HSCT

As of November 30, 2021, the median follow-up time for all patients was 396 (range 340–964) days. No graft failure occurred. The events of GVHD and death of each patient during follow-up were illustrated in Fig. 2. Only 1 patient (PTCy-2) experienced grade II aGVHD on intestine occurring at 115 days post transplantation. There was no grade III or IV aGVHD event. Two patients (PTCy-3, PTCy-8) developed moderate cGVHD on digestive tract and lung, respectively. In addition, 3 patients (PTCy-1, PTCy-2, PTCy-7) had mild cGVHD on liver. No severe cGVHD was observed. Therefore, the 1-year cumulative incidence of moderate-to-severe cGVHD was 25.0% (Fig. 3A).

One patient (PTCy-1) died from diffuse alveolar hemorrhage at 340 days post-transplantation while the other patients were still alive after more than 1-year follow-up. There was no relapse observed. In total, the 1-year OS rate and GRFS rate were estimated to be 87.5% and 62.5%, respectively (Fig. 3B).

## Discussion

Despite the high efficacy for a variety of hematologic malignancies, allo-HSCT is not accessible for many patients due to the absence of HLA-identical donors [27, 28]. Haploidentical donor is an alternative source of graft for nearly all recipients whose biological parents, siblings or offspring are candidates [28]. However, haplo-HSCT is associated with high mortality compared with HLA-matched transplantation, which is mainly caused by elevated rates of GVHD [29]. In recent years, haplo-HSCT with PTCy-based GVHD prophylaxis has been widely used in many hematological malignancies [30]. Yet, there has been few reports on the usage of PTCy regimen in CAEBV patients undergoing haplo-HSCT. Recently, Maegaki M et al. reported successful haplo-HSCT with PTCy in a CAEBV patient who was alive without relapse or severe complications over 1 year after transplantation [31]. Our study, reporting a series of pediatric patients, demonstrates a good performance of preventing GVHD, especially grade III-IV aGVHD and severe cGVHD, and provides preliminary evidence that PTCy may be an alternative choice for GVHD prevention among CAEBV patients treated with haplo-HSCT.

As an emerging strategy for GVHD prevention, PTCy has shown a performance prior to the conventional ATG-based regimen. A retrospective study in unrelated donor HSCT observed reduced cumulative incidences of grade II–IV aGVHD at 100 days and moderate-to-severe cGVHD in PTCy-treated patients compared with ATG-treated patients [22]. Among patients with acute myeloid leukemia (AML) and undergoing mismatched unrelated donor (mMUD) transplantations, PTCy group had significantly lower incidence of 100-day III–IV aGVHD and higher 2-year GRFS than ATG group [32]. Yet, in the setting of CAEBV treated with haplo-HSCT, whether PTCy is better than ATG in terms of GVHD prevention and survival benefit needs more evidence.

There were two main conditioning strategies, including MAC and reduced-intensity conditioning (RIC), before allo-HSCT, both of which yield low incidence of relapse [33]. In allo-HSCT treated AML, there was no difference in terms of OS, cumulative incidence of relapse, cGVHD rate between RIC and MAC, a trend to lower aGVHD rate but an increasing graft failure in RIC [34]. Yet, most of the included studies of this meta-analysis used conventional CNI/MTX combination for GVHD prophylaxis. A recent meta-analysis demonstrated that, when adopting PTCy prophylaxis, MAC was associated with less relapse, better PFS and higher non-relapse mortality, but had no difference in OS, GRFS, grade II-IV aGVHD or cGVHD as compared with RIC [35]. For CAEBV patients who underwent allo-HSCT, a previous study showed superior survival of RIC-treated patients than that of MAC-treated patients [36]. But a recent registry data analysis including 102 systemic CAEBV patients did not find evidence of MAC inferior to RIC [24]. However, most of donors in these reports were HLA-matched, and cord blood and bone marrow were the mostly frequent graft sources. There still lacks evidence comparing MAC and RIC in CAEBV patients treated with haplo-HSCT. In our study, all patients used peripheral blood as graft source and all donors were haploidentical. Considering RIC may reduce engraftment, we chose MAC as conditioning strategy, which resulted in no graft failure or relapse, and low incidence of GVHD.

Despite the worldwide application of PTCy for GVHD prophylaxis, the mechanism of PTCy in preventing GVHD have not been fully elucidated. An in vivo experiment in MHC-haploidentical HSCT murine model demonstrated that PTCy may impair alloreactive T cell function with recovery of regulatory T cells [37]. Moreover, PTCy can eliminate mature NK cells infused with unmanipulated grafts and diminish NK cell alloreactivity [38]. A prospective study has described the immune recovery pace of patients treated with haplo-HSCT and showed a delayed recovery pattern of haploidentical transplantation than HLA-matched transplantation [39]. Nevertheless, the delayed recovery of T-cell subsets and NK cells did not reflect a poor immune reconstitution but a selective depletion of alloreactive T and NK cells [38, 40]. In our study, the counts of T-cell subsets gradually increased within 6 months post HSCT, indicating an immune recovery after haplo-HSCT. More validations are needed for a better understanding of PTCy-induced immune tolerance and identifying the optimal dose of PTCy [41].

Our study has some limitations. First, the sample size is small. Only 8 patients adopting PTCy prophylaxis are included since CAEBV is rare. Secondly, the follow-up is not adequate enough. Thus, some long-term cGVHD events may not be observed and long-term indicators, such 3-year or 5-year survivals and incidences cannot be estimated.

## Conclusion

In conclusion, our study suggests PTCy as an alternative treatment choice for GVHD prophylaxis in CAEBV patients after haplo-HSCT. Further prospective, large-scale trials are needed to confirm these findings.

## Data Availability

The datasets used and/or analysed during the current study are available from the corresponding author on reasonable request.

## Abbreviations

CAEBV: Chronic active Epstein-Barr virus infection
haplo-HSCT: Haploidentical hematopoietic stem cell transplantation
GVHD: Graft-versus-host-disease
PTCy: Post-transplant cyclophosphamide
ATG: Anti-thymocyte globulin
MRD: Matched related donor
MUD: Matched unrelated donor
MAC: Myeloablative conditioning
RIC: Reduced-intensity conditioning
GRFS: GVHD and relapse-free survival
OS: Overall survival
